# Gut Microbiota Dysbiosis in Advanced Chronic Kidney Disease and Kidney Transplantation: A Narrative Review of Pathophysiological Mechanisms, Implications for Nutritional Status, and Biotic Interventions

**DOI:** 10.3390/nu18213502

**Published:** 2026-10-25

**Authors:** Cyntia Carolina García-Ramírez, Fabiola Martín-del Campo, José Efrén Ortíz-González, Tonatiuh González-Heredia, Alfonso M. Cueto-Manzano

**Affiliations:** 1 Medical Research Unit of Renal Disease, Specialties Hospital, Western National Medical Center, Mexican Institute of Social Security, Guadalajara 44320, Mexico; cyntia.garcia4605@alumnos.udg.mx (C.C.G.-R.); fabi_mc@hotmail.com (F.M.-d.C.); jose.ortiz5116@alumnos.udg.mx (J.E.O.-G.); 2 Department of Biomedical Sciences, University Center Tonala, University of Guadalajara, Guadalajara 45125, Mexico; tonatiuh.gonzalez2923@academicos.udg.mx

**Keywords:** kidney transplantation, intestinal microbiome, dysbiosis, probiotics, prebiotics, synbiotics, immune tolerance, uremic toxins

## Abstract

Chronic kidney disease (CKD) is a serious public health issue affecting 10% of the global population. CKD is associated with intestinal dysbiosis, characterized by a reduction in saccharolytic bacteria and an increase in proteolytic genera that produce uremic toxins. This may promote intestinal barrier impairment (“leaky gut” syndrome), chronic low-grade inflammation, and protein-energy wasting (PEW), although most of the supporting evidence is observational. Kidney transplantation (KT) improves survival but may not fully reverse dysbiosis. KT recipients are exposed to residual uremia, use of perioperative antibiotics, and chronic immunosuppressive regimens. Drugs such as mycophenolate mofetil can cause gastrointestinal toxicity and reduced intake due to diarrhea or enteropathy, whereas corticosteroids and calcineurin inhibitors induce hyperphagia, hypomagnesemia, and insulin resistance. These factors may lead to a spectrum of complications ranging from persistent PEW in the early post-transplant period to a higher prevalence of weight gain and obesity, sarcopenia, and post-transplant diabetes. Furthermore, the microbiota may modulate pharmacological responses; in experimental and small clinical studies, genera such as *Faecalibacterium* have been linked to alterations in the metabolism of tacrolimus, potentially altering its therapeutic levels and the risk of nephrotoxicity or rejection. Interventions using biotics (prebiotics, probiotics, and synbiotics) are emerging as a potential adjuvant strategy to restore eubiosis and mitigate PEW. In non-dialysis and dialysis CKD patients, some biotics have been reported to reduce uremic toxins and inflammatory cytokines (IL-6, TNF-α), although results are heterogeneous. However, there is no evidence available regarding its ability to improve nutritional status following a KT. Safety data are limited, although biotic interventions are generally considered safe. Future prospective studies are required to confirm their efficacy and immunological safety, as well as any possible association with infectious adverse events.

## 1. Introduction

Chronic kidney disease (CKD) is a serious public health issue affecting approximately 10% of the global population and is associated with a marked increase in cardiovascular morbidity and mortality, frailty, and functional decline [1]. CKD—particularly in advanced stages (CKD 4–5)—has been linked to gut dysbiosis characterized by a predominance of bacterial families with proteolytic metabolism (producing uremic toxin precursors) and a reduction in saccharolytic genera (producing short-chain fatty acids or SCFAs). This imbalance promotes the systemic accumulation of neurotoxic solutes, impairment of the intestinal barrier, and a chronic low-grade inflammatory state [2]. In turn, these changes have been associated with worsened renal function and the onset of various CKD complications, such as cardiovascular disease, fluid–electrolyte imbalances, cognitive decline, and—importantly—the development or worsening of protein-energy wasting (PEW) [3]. In this context, interventions aimed at modulating the gut microbiota using prebiotics, probiotics, and synbiotics emerge as a promising adjuvant strategy to restore a more eubiotic microbial profile, optimize the production of protective metabolites, and mitigate the systemic complications of CKD.

Kidney transplantation (KT) significantly improves the survival and quality of life for patients with advanced CKD; however, certain alterations persist rather than resolve [4,5]. KT recipients exhibit gut dysbiosis with significant implications, given that the gastrointestinal tract not only harbors the body’s highest density of immune cells, but also possesses a bacterial ecosystem that actively participates in maintaining the epithelial barrier and inducing immunological tolerance through the production of key metabolites, such as SCFAs. This dysbiosis may stem from residual uremia, prophylactic antibiotic therapy, and chronic exposure to immunosuppressants—factors linked to a higher burden of uremic toxins, gastrointestinal complications, and potential metabolic and neurocognitive repercussions. In this context, KT recipients could benefit from microbiota modulation through specific nutritional interventions.

Notwithstanding, the pathophysiological interactions among gut microbiota dysbiosis, nutritional status, and immunosuppressive therapy in patients with advanced CKD and KT have not been completely analyzed. Therefore, this review aimed to approach these issues and to critically evaluate the safety and efficacy of biotic interventions (prebiotics, probiotics, and synbiotics) in modulating the gut–kidney axis in these patients.

A non-systematic literature search was performed in PubMed/MEDLINE, Embase, and the Cochrane Central Register of Controlled Trials from 2009 to 2026, using combinations of the terms “kidney transplantation”, “chronic kidney disease”, “hemodialysis”, “peritoneal dialysis”, “gut microbiota”, “dysbiosis”, “prebiotics”, “probiotics”, “synbiotics”, “nutritional status”, and “immunosuppression”. We included studies in adults with advanced CKD (stages 4–5, including dialysis) or KT recipients that evaluated gut microbiota composition or function, uremic toxins, nutritional status, or the effects of prebiotics, probiotics, or synbiotics. Randomized controlled trials, observational studies, and relevant reviews published in English or Spanish were prioritized, and the reference lists of key articles were also screened. In addition, a specific search for randomized trials of prebiotics, probiotics or synbiotics in dialysis patients reporting nutritional status as an outcome was performed in the same databases (last search: [30 June 2026]); no other trial with this objective was identified.

## 2. Nutritional Status in Chronic Kidney Disease

The nutritional profile of patients with advanced CKD is determined by multiple factors—often present from early stages of the disease—including complications of the underlying pathology and its treatments, genetic and environmental determinants, acute intercurrent episodes, and factors associated with renal replacement therapy (RRT) [3]. Alterations in the nutritional status of these patients are associated with both insufficient nutrient intake and factors such as systemic inflammation, oxidative stress, and hypercatabolism [3]. For this reason, the term PEW has been proposed to replace “classic malnutrition”, since it is not explained by insufficient nutrient intake alone. In addition to various hormonal and metabolic alterations associated with CKD, PEW results from metabolic acidosis, chronic inflammatory state, and RRT itself.

The International Society of Renal Nutrition and Metabolism (ISRNM) proposed diagnostic criteria for PEW that require abnormalities in at least three out of four categories [6]: (i) biochemical indicators (serum albumin < 3.8 g/dL, prealbumin < 30 mg/dL, or total cholesterol < 100 mg/dL); (ii) body mass (BMI < 23 kg/m^2^, unintentional weight loss > 5% in 3 months or >10% in 6 months, or total body fat < 10%); (iii) muscle mass (muscle loss > 5% in 3 months or >10% in 6 months, or a reduction in mid-arm muscle circumference > 10% below the median of the reference population); and (iv) dietary intake (unintentionally low protein intake < 0.80 g/kg/day or low energy intake < 25 kcal/kg/day) [3]. This nutritional abnormality has a high prevalence in CKD—particularly in advanced stages—with rates ranging from 11% to 54% across stages 3 to 5 of the disease, depending on the characteristics of the populations studied, epidemiological and cultural factors, dietary habits, assessment methods, stage of the disease, and the RRT modality [3].

## 3. Evolution of Nutritional Status After Kidney Transplantation

KT, when feasible, represents the best option for patients with advanced CKD. Globally, the frequency of this procedure has steadily increased over recent decades, alongside improvements in surgical techniques and immunosuppressive regimens; this has led to reduced rejection rates and optimized graft and recipient survival [7]. However, the immunosuppressive drugs required to prevent rejection are associated with adverse effects and symptoms that impact nutritional status.

Regardless of the graft source (living or deceased donor), KT can entail clinical complications arising from the immune response to the graft or from the immunosuppressive therapy itself. Notable among these complications are nutritional disorders—including PEW, obesity, sarcopenia, and sarcopenic obesity—as well as fluid–electrolyte and metabolic imbalances and immunosuppression-related gastrointestinal symptoms (Table 1).

Most KT candidates already exhibit nutritional abnormalities; the high prevalence of these issues underscores the need for systematic nutritional assessment prior to transplantation and ongoing monitoring afterward. Many of these abnormalities can worsen during the post-transplant period due to the use of immunosuppressants, episodes of rejection, or increased appetite—driven by both clinical improvement and the relaxation of previous dietary restrictions [17,18].

The reported prevalence of PEW after KT varies widely (approximately 20–52%) [8], probably reflecting differences in the diagnostic criteria and nutritional markers used, the time elapsed since transplantation, and the characteristics of the populations studied. Conversely, weight gain is common after KT, although its frequency depends on how it is defined. During the first year after transplantation, it was reported in 54.6% of recipients in a single-center Saudi cohort, using an increase in body mass index ≥2 kg/m^2^ [10], and in 72.7% in a Brazilian cohort, where any increase in body weight was counted (mean gain 7.12 ± 5.9 kg) [11]. Moreover, obesity became more frequent in a single-center study in Italy including 96 recipients evaluated on average 7.2 years after transplantation, with obesity defined as a body mass index ≥ 30 kg/m^2^; its prevalence rose from 6.3% at transplantation to 18.8%, an approximately threefold increase, with greater weight gain in women than in men [9]. During the immediate post-transplant period (<6 weeks), nutritional demands are high due to the immunometabolic response to surgical trauma and the use of high-dose immunosuppressants, both of which promote protein catabolism. During this time, it is essential to maintain adequate nutritional status, promote wound healing and anabolism, and minimize the risk of infection and graft rejection [19]. The late post-transplant period (>6 weeks) is characterized by recovery, including metabolic normalization, improved quality of life, reduced nutritional requirements, lower medication dosages, and better management of adverse drug effects [19]. Nutritional monitoring is crucial during this phase to adjust caloric intake and prevent excessive energy consumption that could lead to overweight or obesity and their associated complications, such as impaired graft function and increased cardiovascular risk [20].

## 4. Gut Microbiota in Kidney Disease

### 4.1. Pre-Existing Uremic Dysbiosis

In a state of eubiosis, the gut maintains a predominance of saccharolytic bacteria (primarily from the *Firmicutes* and *Bacteroidetes* phyla, notably the *Bifidobacterium* and *Lactobacillus* genera) that ferment complex carbohydrates and produce SCFAs [21]. However, the retention of uremic toxins in advanced chronic kidney disease radically alters this microenvironment (Figure 1). Through the fermentation of aromatic amino acids, gut bacteria generate precursors such as indole (from tryptophan) and p-cresol (from tyrosine and phenylalanine); these are absorbed and conjugated by the host into indoxyl sulfate (IS) and p-cresyl sulfate (pCS), respectively [21]. Phenylacetylglutamine (PAG) results from the glutamine conjugation of phenylacetic acid, which derives almost exclusively from the microbial conversion of phenylalanine [22]. In a separate pathway, gut bacteria convert dietary choline into trimethylamine, which is oxidized by hepatic flavin-containing monooxygenase 3 (FMO3) into trimethylamine N-oxide (TMAO) [23]. Lipopolysaccharide (LPS), in contrast, is a structural component of the outer membrane of Gram-negative bacteria that is released into the intestinal lumen via bacterial lysis or translocation, rather than being a direct product of fermentation. At the level of the renal tubular epithelium, the organic anion transporter mediates the uptake of these toxins, increasing the production of nicotinamide adenine dinucleotide phosphate (NADPH) and reactive oxygen species (ROS) [24]. IS activates the aryl hydrocarbon receptor (AhR) pathway and nuclear factor-kappa B (NF-κB), increasing the expression of cytochrome P450 and inflammatory cytokines [25]; TMAO likewise activates NF-κB, increasing interleukin-6 (IL-6) and the nucleotide-binding oligomerization domain, leucine-rich repeat and pyrin domain-containing protein 3 (NLRP3) inflammasome [26]. At the intestinal level, LPS disrupts intestinal epithelial tight junction proteins—particularly occludin and claudin-1—leading to “leaky gut”. These proteins are inversely correlated with circulating endotoxemia; this disruption facilitates the translocation of viable bacteria and bacterial products into the systemic circulation, thereby contributing to endotoxemia and chronic low-grade inflammation [27]. Saccharolytic microbiota generates protective metabolites, including SCFAs and indole-3-propionic acid (IPA). IPA acts via the pregnane X receptor (PXR) and the AhR to limit intestinal inflammation [28,29] and stimulates intestinal L-cells to produce glucagon-like peptide-1 (GLP-1) [30]. Of note, AhR and PXR are ligand-activated receptors through which microbial indole derivatives signal (PXR being a nuclear receptor), whereas NLRP3 is a cytosolic inflammasome complex activated downstream of TMAO, not a receptor for these ligands. Dysbiosis and the resulting accumulation of uremic toxins have a negative systemic impact and contribute to progressive kidney injury [21,31]. Beyond the kidney, uremic toxins and the inflammatory status derived from dysbiosis have also been linked to cognitive status impairment through the gut–brain–kidney axis, as they may cross and disrupt the blood–brain barrier and promote neuroinflammation [1,2].

Impaired renal clearance causes a massive influx of urea into the intestinal lumen via enterohepatic circulation [21]. There, urease-producing bacteria hydrolyze urea into ammonia (NH_3_) and subsequently ammonium hydroxide (NH_4_OH), significantly raising the luminal pH [21,31]. This drastic pH shift has an adverse effect: it destroys ecological niches of saccharolytic bacteria and promotes the overgrowth of bacterial families with proteolytic metabolism, such as Enterobacteriaceae, Pseudomonadaceae, and Clostridiaceae [31].

In addition to low food intake, patients with CKD are exposed to numerous factors that promote malnutrition and PEW, including endocrine disturbances, cardiovascular and metabolic comorbidities, dialysis-related factors, metabolic acidosis, chronic inflammation, and oxidative stress [3]. Dysbiosis induced by inadequate dietary patterns (particularly low fiber intake) may contribute to the development of PEW [32]. However, available evidence remains limited, and the implications of gut microbiota alterations in the pathogenesis of PEW must be interpreted with caution [32]. Microbiota modulation via dietary fiber, prebiotics, and probiotics constitutes the pathophysiological basis for the nutritional interventions discussed later. Furthermore, in some patients with advanced CKD—whether on dialysis or not—dietary restrictions aimed at controlling serum potassium may inadvertently reduce fiber intake, because several potassium-containing foods (fruits, vegetables, legumes, and nuts) also provide dietary fiber [20]. Reduced fiber consumption has been described as a contributing factor to dysbiosis in uremia [21] and may deprive commensal species of fermentation substrates (microbiota-accessible carbohydrates). Potassium and fiber restriction are, however, not equivalent: dietary potassium should be adjusted to keep serum potassium within the normal range and tailored to each patient’s individual needs, considering the overall diet [20]; accordingly, strict fiber limitation should not be assumed for all patients with advanced CKD. Dietary recommendations should therefore be individualized to each patient’s biochemical profile, comorbidities, and tolerance, aiming to preserve fiber intake whenever possible. Consequently, it is possible that some patients may arrive at KT surgery with a depleted, pro-inflammatory microbiota and a reduced capacity to synthesize SCFAs [21].

### 4.2. Microbial Profile and Intestinal Function in Kidney Transplantation

Following KT, the gut microbiota exerts complex local and systemic effects, participating in metabolic and immune processes, among others [33]. A cross-sectional study comparing the composition and function of the microbiota among KT recipients, CKD patients on hemodialysis, and healthy subjects [34] found that the microbial profiles of transplant recipients and hemodialysis patients were similar to each other but differed significantly from those of healthy controls; specifically, the abundance of Firmicutes, Lachnospiraceae, Ruminococcaceae, and *Faecalibacterium* was significantly lower, whereas Bacteroidetes, Proteobacteria, Clostridiales, and Enterobacteriaceae were increased in both patient groups compared to healthy subjects. Furthermore, certain genera within the Lachnospiraceae and Veillonellaceae families showed a negative correlation with clinical markers such as serum creatinine and blood urea nitrogen [34]. These changes in the microbiota of transplant patients have been linked to functional alterations, including increased efficiency in carbohydrate metabolism and a reduction in pathways related to cofactor and vitamin metabolism, cell motility, and genetic information processing [34]. In the same study, functional analysis showed enhanced carbohydrate metabolism, including glycolysis/gluconeogenesis, in transplant recipients compared with CKD patients, and *Roseburia*, a genus of the family Lachnospiraceae, was among the taxa depleted in recipients relative to healthy controls [34]. Consistently, in a study of 25 Chinese living kidney donors (mostly first-degree relatives) and their recipients, the gut microbiota of recipients before transplantation was similar to that of their donors, whereas samples obtained within the first two months after transplantation showed a distinct community structure with lower alpha diversity, a higher relative abundance of *Escherichia–Shigella* and *Streptococcus*, and a decrease in *Roseburia* [35].

Another study longitudinally analyzed the gut microbiota of KT patients at three time points: pre-transplantation, and 7 and 30 days post-KT. A decrease in microbial abundance was demonstrated in post-transplant samples compared to the baseline samples. Certain bacterial groups—such as Clostridiales, Clostridia, Ruminococcaceae, *Faecalibacterium*, and Veillonellaceae—were significantly more abundant prior to KT, whereas Bacilli, Enterococcaceae, and *Enterococcus* increased post-KT, particularly at the 30-day mark [33]. These changes are not merely quantitative but also functional and clinically relevant. Various Clostridia bacteria degrade bile acids, bolster intestinal barrier immune defenses, and modulate inflammation, whereas species of the genus *Enterococcus* are associated with opportunistic infections; in KT recipients, higher fecal abundance of *Enterococcus* has been linked to an increased risk of urinary tract infection (24% vs. 0% in patients without such abundance; *p* = 0.005), and its expansion (along with that of *Escherichia* and *Lachnoclostridium*) has also been observed in patients with post-transplant diarrhea [5]. A notable example is *Faecalibacterium*—specifically *Faecalibacterium prausnitzii*—a key producer of butyrate [36]. Its depletion has been linked to inflammatory bowel disease, and as detailed later, to the pharmacokinetics of immunosuppressive drugs in KT. Table 2 summarizes the main taxonomic changes described following a KT.

Importantly, there may be multiple reasons for the discrepancies between the studies described above [33,34,35,37,38]. First, most of the studies are small, single-center and observational; second, sequencing strategies differ (different 16S rRNA hypervariable regions and platforms were used), which affects taxonomic resolution and relative abundances; third, sampling and control groups vary, with single versus serial time points; fourth, different antibiotic exposure; fifth, immunosuppressive regimens are heterogeneous. Finally, dietary intake, a recognized determinant of gut microbial composition, was not systematically assessed in most of these studies. Taken together, these differences limit comparability, and the taxonomic changes shown in Table 2 should be interpreted as a trend rather than established microbial signatures.

**Table 2:** Altered gut microbiota taxa in chronic kidney disease and after kidney transplantation.

Taxon (Level)	Direction of Change	Functional/Clinical Significance
Firmicutes (phylum), Lachnospiraceae, Ruminococcaceae (families)	Decreased	Loss of key SCFAs-producing taxa; reduced availability of butyrate for colonocyte metabolism and immune regulation [34,38].
*Faecalibacterium* (specially *F. prausnitzii*)	Decreased	Key butyrate producer; its reduced abundance is associated with compromised mucosal integrity, whereas a higher abundance has been associated with higher tacrolimus requirements [37,39].
Bacteroidetes (phylum)	Increased	Relative expansion accompanying loss of Firmicutes dominance; alteration of carbohydrate fermentation capacity [34].
Proteobacteria (phylum), Enterobacteriaceae (family)	Increased	Overgrowth of proteolytic and pro-inflammatory taxa favored by increased luminal pH and urea flux; associated with endotoxemia and systemic inflammation [21,31].
*Enterococcus* (genus)	Increased early post-transplant (vs. pre-transplant)	Expansion due to perioperative antibiotic exposure; associated with risk of opportunistic infection in the immediate post-transplant period [5,33].
*Roseburia* (genus)	Decreased vs healthy controls; decrease after KT not significant after multiple-testing adjustment	Loss of a butyrate-producing Lachnospiraceae [34,35].

SCFAs: short-chain fatty acids. Directions of change are relative to healthy controls unless otherwise specified.

### 4.3. The Ecological Cost of Pharmacological Therapy

Standard perioperative management for KT requires short-course prophylactic antibiotic therapy (a first-generation cephalosporin is the most broadly used, i.e., cefazoline) to prevent surgical site infections [37,38]. Long-term or extended antibiotic prophylaxis in KT recipients is primarily used to prevent opportunistic infections, and the preferred antibiotic is trimethoprim-sulfamethoxazole [37,38]. Treatment of established infections (e.g., third-generation cephalosporins or piperacillin/tazobactam) depends on the type of infection and isolated agent. Depending on class, duration, and cumulative exposure, these agents can deplete susceptible commensal bacteria and reduce alpha diversity [40,41]. The depletion of anaerobic bacteria leaves nutrients available and favors the expansion of opportunistic pathogens [40]. Multiple metagenomic studies using 16S rRNA gene sequencing demonstrate that in the first few weeks post-transplantation, there is a critical contraction of butyrate-producing genera (such as *Faecalibacterium, Roseburia*, and *Coprococcus*) and an exponential expansion of potentially pathogenic taxa—such as *Enterococcus* (particularly vancomycin-resistant strains) and *Clostridioides difficile*—thereby increasing the risk of bacteremia and sepsis [39]. This phenomenon is not exclusive to transplantation. Infections are frequent in advanced CKD even before RRT: in a nationwide cohort of patients with advanced CKD who started dialysis, 32.7% had at least one infection prior to dialysis initiation [42]. In patients undergoing hemodialysis, infection-related morbidity and mortality remain higher than in the general population [43], and catheter-related infections are commonly managed with broad-spectrum empirical antibiotics such as vancomycin combined with aminoglycosides [44]. Repeated antibiotic exposure can further reduce microbial diversity, eliminate commensal taxa, and favor antibiotic-resistant strains, thereby compounding the pre-existing uremic dysbiosis [45].

### 4.4. Immunosuppressants as Modulators of the Digestive Ecosystem

Once the immediate post-surgical phase has passed, maintenance immunosuppression—predominantly involving a combination of a calcineurin inhibitor (CNI) such as tacrolimus or cyclosporine, an antiproliferative agent like mycophenolate mofetil (MMF), and corticosteroids—exerts continuous selective pressure on the gastrointestinal tract [46].

CNIs may possess direct antimicrobial activity, since tacrolimus is a macrolide originally developed as an antibiotic, although its direct effect on specific gut bacteria has not been studied [47]. By inhibiting the calcineurin/nuclear factor of activated T-cells (NFAT) pathway, these drugs not only suppress host T-cells, but may also alter digestive epithelial defense mechanisms, such as the secretion of antimicrobial peptides (alpha-defensins) by Paneth cells and mucus production by goblet cells [47]; these mechanisms are largely hypothetical, with experimental evidence limited to reduced expression of the antimicrobial lectins RegIIIβ and RegIIIγ in mice [47]. In animal models, tacrolimus has been associated with a decline in bacterial taxa such as Ruminococcaceae [46,47]; whether this occurs in KT recipients has not been established.

MMF is the immunosuppressive drug most closely linked to direct gastrointestinal toxicity. By blocking the de novo synthesis of guanosine nucleotides, it severely affects rapidly replicating cells, such as enterocytes. MMF has been associated with intestinal villous denudation and mucosal inflammation resembling Crohn’s disease. This MMF-induced enteropathy exacerbates dysbiosis by causing profound alterations in microbial composition, drastically reducing the abundance of commensal bacteria and promoting a local hyperinflammatory state [14,47].

### 4.5. Bidirectional Interaction Mechanisms

Some studies suggest that the efficacy and safety of post-transplant immunosuppressive therapy may be influenced by the recipient’s bacterial composition. Tacrolimus has an extremely narrow therapeutic window and highly variable oral bioavailability. Certain species—particularly those of the genus *Faecalibacterium* (e.g., *Faecalibacterium prausnitzii*)—have been shown in vitro to metabolize tacrolimus, converting it into M1 (9-hydroxy-tacrolimus), a metabolite approximately 15-fold less potent than the parent drug [48]. In a small pilot study, a higher relative abundance of *Faecalibacterium* in the gut microbiota was associated with higher tacrolimus dose requirements to achieve therapeutic blood levels [37]. Based on these observations, it has been hypothesized that if the microbial composition changes abruptly (e.g., due to antibiotic use), the rate of tacrolimus biotransformation could decrease, potentially raising the serum levels and increasing the risk of nephrotoxicity; conversely, excessive proliferation of these metabolizing bacteria could reduce the serum levels below the therapeutic range, potentially increasing the risk of acute T-cell-mediated rejection [37,48,49]. However, these scenarios remain speculative and have not been demonstrated in clinical studies. The available evidence derives mainly from a single small observational cohort (19 recipients) [37] and from *in vitro* work [48], with no interventional data, and tacrolimus exposure is also influenced by other factors such as CYP3A5 genotype, diet, concomitant drugs, and hematocrit [50,51].

### 4.6. Metabolism of Uremic Toxins of Bacterial Origin

At the cellular level, in experimental models, the binding of IS and pCS to proximal tubular cells activates the intrarenal renin–angiotensin–aldosterone system, increasing the expression of renin, angiotensinogen, and the AT1 receptor while reducing that of the AT2 receptor [52]. In these same models, activation of such systems leads to a sustained increase in transforming growth factor-beta 1 (TGF-β1) and the phosphorylation of SMAD2 and SMAD3 proteins, which act as intracellular effectors of its signaling pathway. Consequently, tubular cells lose epithelial markers (such as E-cadherin) and acquire mesenchymal characteristics—fibronectin and alpha-smooth muscle actin—a phenomenon known as epithelial–mesenchymal transition that manifests histologically as interstitial and glomerular fibrosis [52]. These findings derive from experimental models of kidney injury and have not been demonstrated in KT; their relevance to graft fibrosis, therefore, remains to be established. This same axis involving uremic toxins and oxidative stress has been proposed to contribute to vascular wall calcification and systemic endothelial dysfunction; these mechanisms may explain why the accumulation of IS and pCS may compromise not only graft survival, but also the cardiovascular risk of the KT recipient [53].

## 5. Evidence of the Effect of Nutritional Interventions with Biotics

The data summarized below pertain to populations with non-dialysis CKD, hemodialysis, peritoneal dialysis and KT; therefore, they are not necessarily extrapolable from one group to another.

### 5.1. Probiotics in Chronic Kidney Disease and Kidney Transplantation

Recently, probiotics have been proposed as a complementary intervention for CKD, aiming to modulate the gut microbiota and reduce the production and absorption of bacterial-derived uremic toxins, primarily pCS and IS. The formulations studied are largely based on combinations of *Lactobacillus* species (*L*. *acidophilus*, *L*. *casei*, *L*. *plantarum*, *L*. *rhamnosus*, among others) and *Bifidobacterium* species (*B*. *longum*, *B*. *bifidum*), sometimes combined with *Streptococcus thermophilus* or *Bacillus coagulans*. Dosages typically range from 10^7^ to 10^11^ colony-forming units (CFU)/day, with treatment durations spanning from 4 weeks to 6 months. In non-dialysis CKD (stages 3–4), two multi-strain probiotic combinations (Lobun Forte™ and Renadyl™) were compared over six months in 60 patients (30 per group) in a randomized, open-label, active-controlled trial without a placebo arm and with similar baseline characteristics between groups [54]. The Renadyl™ group showed a significant reduction in both pCS (21%; *p* = 0.03) and IS (24%; *p* < 0.0001), whereas the Lobun Forte™ group significantly reduced IS (30%; *p* < 0.0001) but not pCS (8%; *p* > 0.05), illustrating the heterogeneity of response even among partially similar formulations. Both groups also demonstrated significant improvements in eGFR, quality of life, and BUN and creatinine levels; however, these were within-group changes in a small, short, industry-sponsored trial without a placebo comparator, so their clinical relevance should be interpreted with caution.

In the context of hemodialysis, one randomized, double-blind, placebo-controlled study [55] evaluated the use of a single-strain *Lactobacillus rhamnosus* probiotic over four weeks in 42 patients (21 per group) with comparable baseline toxin levels, finding a significant reduction in phenol and p-cresol compared to the placebo after adjustment for baseline values; this study did not include IS among its outcomes, and its short duration and surrogate endpoints (serum toxins, not clinical outcomes) limit its clinical relevance. Liu et al. evaluated a multi-strain probiotic over 6 months in a randomized, double-blind, placebo-controlled trial in which 50 patients were randomized and 45 completed follow-up (16 with type 2 diabetes), using fecal microbiota sequencing. Although no significant changes in overall species diversity were found, a restoration of bacterial community composition was observed only in the non-diabetic subgroup (*p* = 0.007), characterized by an increase in the Bacteroidaceae and Enterococcaceae families and a reduction in Ruminococcaceae, Halomonadaceae, Peptostreptococcaceae, and Clostridiales family XIII [56]. In the context of peritoneal dialysis, another study [57] evaluated a combination of four strains (*Bifidobacterium bifidum*, *B*. *catenulatum*, *B*. *longum*, and *Lactobacillus plantarum*) against a placebo over six months in 39 patients who completed the trial (21 vs. 18); the study observed a significant reduction in endotoxin, TNF-α, and IL-6 levels, an increase in IL-10, and better preservation of residual renal function, with no clear differences reported in the incidence of peritonitis. These were mainly surrogate biomarkers, and the available report does not allow for an assessment of baseline differences between groups.

Unlike the cases of non-dialysis CKD, hemodialysis, or peritoneal dialysis, controlled clinical trials evaluating a probiotic alone in KT recipients are notably scarce. This limitation is not unique to the renal population; a Cochrane review focusing on solid organ transplantation as a whole identified only five randomized controlled trials (involving 250 participants) regarding any type of biotic intervention—with only one involving KT recipients—and concluded that the evidence is insufficient to determine the efficacy of these interventions [58]. A retrospective cohort study using propensity score matching [59] included 354 KT recipients, 97 (27%) of whom received probiotics (predominantly *Lactobacillus* species [45%] and *Bacillus subtilis* [14%]) during the first year post-transplant. No significant differences in eGFR were observed at 1 or 3 years between the groups receiving probiotics and those that did not, nor were there differences in the incidence of acute rejection, graft failure, or bacterial infections. However, probiotic supplementation was independently associated with a higher incidence of cytomegalovirus viremia (26% vs. 14%; adjusted odds ratio 2.32; 95% CI 1.21–4.45; *p* = 0.011)—an association that was more pronounced among those who used probiotics for more than three months. However, this study did not provide details regarding the pre-transplant cytomegalovirus serological risk in the recipients, nor the immunosuppression doses used during rejection episodes in either group; furthermore, propensity score matching only allows for the control of measured covariates. This was a retrospective study; therefore, it is not possible to establish a causal relationship. This finding offers a perspective distinct from that of trials involving synbiotics and prebiotics—which have focused primarily on outcomes related to uremic toxins or gastrointestinal symptoms—and signals a potential risk associated with probiotic use. Nevertheless, overall, the current evidence suggests that probiotic use in KT may be beneficial but should be approached with caution, highlighting the need for larger prospective studies to evaluate both efficacy and safety profiles before routine use is recommended.

### 5.2. Prebiotics in Chronic Kidney Disease and Kidney Transplantation

Prebiotics serve as a complementary tool for modulating gut microbiota by providing selective fermentable substrates, particularly inulin and fructooligosaccharides (FOSs).

None of the trials involving non-dialysis CKD patients showed significant changes in eGFR or serum creatinine during the study follow-up periods, nor did they yield conclusive data regarding the initiation of dialysis, hospitalizations, or mortality, due to limited sample sizes and short study durations. The use of high-amylose resistant starch in 75 patients with early-stage diabetic nephropathy was not associated with a significant reduction in urea levels at 12 weeks compared to the control group [60]; that same study found no relevant differences in C-reactive protein (CRP) or IL-6 levels with the use of resistant starch. Trials that included microbiota analysis report heterogeneous results depending on the intervention modality and type. In the context of non-dialysis CKD, Sohn et al. conducted a three-phase pilot study—neither randomized nor blinded—evaluating the effect of oligofructose-enriched inulin (p-inulin) over 12 weeks using 16S rRNA sequencing of serial fecal samples; they documented a significant increase in the abundance of *Bifidobacterium* and *Anaerostipes* during treatment [61].

In hemodialysis settings, several trials have evaluated inulin or resistant starch as an adjunct to standard care. A randomized clinical trial involving 44 hemodialysis patients [62] reported a significant reduction in serum p-cresol levels after 8 weeks of supplementation with high-amylose maize-derived type 2 resistant starch (HAM-RS2) (*p* = 0.04), with no significant changes in IS levels. Regarding inflammation, significant reductions in IL-6, oxidative stress, and IS levels have been reported following resistant starch supplementation in hemodialysis patients [63]. Another study reported that fermentable fiber improved the lipid profile and oxidative status, and reduced systemic inflammation [64]. No effects on serum albumin, nutritional status indicators, or major clinical parameters (residual kidney function, hospitalizations, mortality) were observed in any of these trials. In peritoneal dialysis, the administration of green banana flour (48% resistant starch) for 4 weeks in a crossover trial of 43 randomized patients (26 completed) did not result in improved serum levels of IS, pCS, or indole-3-acetic acid compared to the placebo; a slight increase in IL-6 was observed following supplementation, which the authors did not attribute to the intervention, since it did not differ from the placebo [65]. A different research group, in a series of publications regarding the same cohort of continuous ambulatory peritoneal dialysis patients treated with inulin-type fructans for 12 weeks, reported more encouraging findings regarding the microbiota: supplementation significantly reduced serum uric acid by enriching purine-degrading bacteria (Clostridiales and *Clostridium* species), which increased fecal uric acid degradation [66]; it also increased the Firmicutes/Bacteroidetes ratio (*p* = 0.049) and limited the rise in microbiota-generated indole, although it did not significantly reduce plasma TMAO [67,68]. Taken together, these findings suggest that prebiotics can modify gut microbiota composition in CKD, although the exact pattern of change varies considerably depending on the RRT modality, the type of intervention, and the population studied, with no consistent or replicated pattern across studies. These differences may reflect the type of fiber, which varies in fermentability (inulin-type fructans [67], resistant starch [62,63], banana flour [65], soluble fiber [64]) and the dose and duration of the intervention, which ranged from 10 g/day for 12 weeks [67] to 21 g/day for 4 weeks [65]; in the latter, the IS reduction appeared only in participants who consumed doses close to those proposed [65]. The dietary background, usually the habitual diet and low in fiber in dialysis patients (about 7.6 g/day in one trial [67]), and attrition in crossover trials (26 out of 43 and 15 out of 21 completed [65,67]) may also contribute.

Meanwhile, experience with prebiotics in KT recipients comes from two randomized clinical trials. One of these evaluated a green banana resistant starch supplement over 7 weeks (7.5 g/day for 2 weeks, then 15 g/day) in 56 KT recipients, finding a significant reduction in gastrointestinal symptoms compared to the placebo, with no difference in infection rates (33% vs. 34%) [69]. In a subsequent publication regarding the same cohort—focusing specifically on the microbiota—the same group reported a significant increase in microbial richness and a trend toward increased Shannon diversity in the treated group [70]. The second trial was a randomized, double-blind, placebo-controlled, multicenter study conducted in Australia that evaluated inulin (10 g/day for one week, then 20 g/day) for 4 weeks before and after a third dose of the SARS-CoV-2 vaccine in 72 transplant recipients (37 receiving inulin, 35 receiving placebo); supplementation was feasible, well-tolerated, and safe, and—based on functional prediction using PICRUSt2—led to a significant increase in intestinal *Bifidobacterium* and in KEGG metabolic pathways associated with SCFA production, although it failed to improve live-virus neutralization *in vitro* following vaccination [71]. Regarding safety, both trials reported a favorable profile: the most frequent adverse effects were mild-to-moderate gastrointestinal symptoms (abdominal bloating, flatulence and changes in bowel movement frequency), which in some cases led to dose adjustments, but without serious complications directly attributable to resistant starch or inulin. With only two trials available—involving different populations and objectives—the evidence regarding the use of prebiotics in RRT remains insufficient.

### 5.3. Synbiotics in Chronic Kidney Disease and Kidney Transplantation

Synbiotics combine probiotics and prebiotics in a single formulation to enhance and stabilize the modulation of gut microbiota. At least four controlled trials involving synbiotics have been identified in non-dialysis CKD. A pilot trial involving patients with stage G3b–G4 CKD evaluated a formulation containing *Lactobacillus casei* and *Bifidobacterium animalis* combined with FOSs over an 8-week period; the study observed a decrease in free serum IS and intestinal permeability, as well as an improvement in gastrointestinal symptoms [72]. The SYNERGY trial evaluated a multi-strain synbiotic (9 probiotic strains plus 15 g/day of a mixture of FOS, galactooligosaccharides, and inulin) over 6 weeks in 37 patients with pre-dialysis stage 4 CKD; findings included a 22–28% reduction in IS and pCS, an increase in the fecal abundance of *Bifidobacterium* and Lachnospiraceae, a decrease in Clostridia, and improved se rum albumin levels [73]. Other researchers evaluating a synbiotic in stage G3b CKD patients over 6 weeks—focusing on urea and creatinine outcomes—found no significant differences compared to the placebo [74]. More recently, a double-blind trial in Serbia evaluating the effect of a synbiotic on uremic toxins, inflammation, and the gut microbiome in non-dialysis CKD found no significant effects on BUN or potassium levels [75]. Results regarding the effect of synbiotics on inflammatory markers, such as CRP, have been inconsistent [67,68]. Furthermore, none of the aforementioned trials demonstrated consistent impacts on eGFR or major clinical events (initiation of dialysis, hospitalization, mortality), likely due to their short duration and limited sample sizes.

In the context of hemodialysis, synbiotics have been the most extensively studied method of microbiota modulation. In a study involving 99 patients, the use of a synbiotic food (pasteurized milk with *Bifidobacterium longum* vs. extruded sorghum as a prebiotic) resulted in reported significant reductions in serum concentrations of uremic toxins compared to the placebo [76]. Meanwhile, a controlled trial involving 60 hemodialysis patients found no significant differences in IS levels between the synbiotic and placebo groups (*p* = 0.44), although there was significant improvement in constipation and associated quality of life (*p* = 0.006 and *p* = 0.001, respectively); however, this trial did not include an analysis of fecal microbiota [77]. These findings illustrate that the effect of synbiotics on uremic toxins in hemodialysis is not uniform, yet clinically relevant benefits may occur independently of biochemical changes in IS or pCS.

In the context of KT, two controlled trials involving synbiotics were identified. Guida et al. evaluated a multi-strain synbiotic (*Lactobacillus*, *Bifidobacterium*, and *Streptococcus thermophilus* combined with inulin and resistant starch) against a cellulose control over a 30 days period in stable transplant recipients (more than 12 months post-transplant, with stable graft function. The synbiotic significantly reduced the plasma pCS levels from baseline by 40% and 33% after 15 and 30 days of treatment, respectively (both *p* < 0.05), whereas levels remained stable in the control group, with no significant changes observed in renal function. It should be noted that borborygmi were reported as occasional by most patients (60%) in the synbiotic group, whereas abdominal pain was virtually absent in both groups; the authors considered the treatment well-tolerated, with no relevant gastrointestinal adverse effects [78]. More recently, another commercial synbiotic (LactoCare™)—containing 12 strains from the genera *Lactobacillus*, *Bifidobacterium*, and *Streptococcus thermophilus* alongside FOSs as the prebiotic component—was evaluated over 12 weeks in 46 overweight or obese KT recipients. This study took a different approach, focusing on intestinal permeability and systemic inflammation: the synbiotic group showed significant reductions compared to the placebo in LPS-binding protein (*p* = 0.03), high-sensitivity CRP (*p* = 0.02), soluble intercellular adhesion molecule-1 (*p* = 0.04), and IL-6 (*p* = 0.02), with no significant changes in serum urea or creatinine levels [79]. It is important to note that none of these trials included a direct analysis of gut microbiota composition; thus, none provided empirical evidence regarding which specific taxonomic changes might mediate the observed effects. Given the limited number of available trials, the results should be interpreted with caution. Regarding safety, the synbiotic primarily caused mild gastrointestinal effects (flatulence, abdominal bloating, changes in bowel habits) of a magnitude similar to that reported with probiotics and prebiotics administered in isolation to this same population. To date, no serious infections directly attributable to the probiotic strains have been documented; however, given the small sample size, the ability to detect rare adverse events is limited.

It is worth noting that our group has recently published preliminary results of the only randomized, double-blind, parallel-group clinical trial to date comparing the administration of a prebiotic (20 g of soluble blue agave inulin), a probiotic (20 probiotic bacterial strains: *Bacillus coagulans*, *Bacillus subtilis*, *Bifidobacterium bifidum*, *Bifidobacterium breve*, *Bifidobacterium infantis*, *Bifidobacterium longum*, *Lactobacillus acidophilus*, *Lactobacillus brevis*, *Lactobacillus casei*, *Lactobacillus gasseri*, *Lactobacillus helveticus*, *Lactobacillus paracasei*, *Lactobacillus plantarum*, *Lactobacillus rhamnosus*, *Lactobacillus salivarius*, *Lactococcus lactis*, *Pediococcus acidilactici*, *Pediococcus parvulus*, *Weissella confusa*, and *Weissella paramesenteroides* [dose of 2 × 10^8^ CFU] from a commercial freeze-dried supplement), and a synbiotic (2 × 10^8^ CFU of probiotic bacteria + 20 g of prebiotic fiber [inulin]) against a placebo (maltodextrin) in patients undergoing automated peritoneal dialysis, in order to evaluate their effects on nutritional status. A significant improvement in nutritional status (assessed via Subjective Global Assessment) was observed in the group receiving the prebiotic, as well as a marginal improvement in the group treated with the probiotic compared to the placebo; furthermore, a more pronounced reduction in the percentage of patients with PEW was noted in the group receiving the prebiotic compared to the other groups [80]. As of now, these promising results can be considered as preliminary.

Taken together, these findings come from heterogeneous populations and cannot be extrapolated between them. Very few of the studies reviewed reported nutritional outcomes, and those that did it were small, short and largely negative or inconclusive (e.g., no effects on serum albumin or nutritional status indicators in hemodialysis trials [62,63,64]). Most of the benefits reported were changes in uremic toxins, inflammatory markers, or microbiota composition, which are surrogate outcomes and do not necessarily translate into improvements in PEW, muscle mass, physical performance, or dietary intake; the latter two aspects were seldom evaluated. The only trial designed to evaluate nutritional status as its main objective was conducted in peritoneal dialysis and is available only as a doctoral thesis and abstract [80]. In KT recipients, no trial was designed to primarily evaluate nutritional status. Body weight and dietary intake were reported only as secondary outcomes in one trial of overweight or obese recipients on a calorie-restricted diet; body weight decreased in both groups under a calorie-restricted diet, with a greater reduction in the synbiotic group (−2.0 vs. −0.8 kg; *p* = 0.02), without differences in dietary intake [79].

The current evidence is therefore, although promising, currently insufficient to support the use of biotics to optimize nutritional status in advanced CKD or KT.

### 5.4. The Debate on Clinical Safety in the Immunosuppressed Patient

The use of live microorganisms in immunosuppressed patients (especially during the first few months post-transplant) has been a subject of intense debate and medical caution [81]. The primary theoretical risk is the possibility that a probiotic strain might cross a compromised intestinal barrier and—taking advantage of the host’s suppressed immune status—enter the bloodstream, causing bacteremia, fungemia, or sepsis [40,81]. Controlled clinical trials and prospective cohort studies involving solid-organ transplant recipients report that the incidence of such serious adverse events is statistically indistinguishable from that observed with a placebo [58,81]; however, these studies were small and of short follow-up, and the certainty of the evidence is very low [58], so rare bloodstream infections or other serious adverse events cannot be reliably excluded. Reported cases of *Lactobacillus* sepsis are linked almost exclusively to critically ill patients in intensive care units who have central venous catheters or severe short-bowel syndrome—scenarios different from that of the outpatient KT recipient, although this does not exclude the risk in the early post-transplant period [81,82]. Safety recommendations for live-organism probiotics in immunosuppressed patients are largely extrapolated from non-transplant populations. Prebiotics, being non-viable substrates, do not carry the risk of bacterial translocation, whereas probiotics and synbiotics contain live microorganisms; if used, these latter products should meet strict quality criteria: strains must be fully identified (including at the genotypic level) and free of transferable antibiotic-resistance plasmids; administration should occur during periods of immunological stability (preferably after the immediate post-transplant phase has passed) and under the guidance and close monitoring of a multidisciplinary nephrology and clinical nutrition team [58,82]. Although a previous retrospective study has indicated a possible association between probiotic use in KT and viral infections, prospective studies with better methodological designs and larger sample sizes are needed to address this issue more adequately.

### 5.5. Effect of Biotic Interventions on Gut Microbiota Composition in Kidney Transplantation

Beyond the clinical and biochemical outcomes described, a fundamental goal of biotic intervention is to directly correct dysbiosis in transplant recipients [4,5]. However, evidence regarding the direct use of biotics to correct these alterations in the transplant population is virtually non-existent (Table 3): of the four available randomized controlled trials involving transplant recipients (two using prebiotics and two using synbiotics), only one included a direct outcome measure related to microbiota composition [70]. No randomized controlled trials investigating the exclusive use of probiotics in transplant recipients were identified; the only available study on probiotics [59] is retrospective and observational in design, rather than a randomized controlled clinical trial. A summary of the design, sample size, intervention, duration and main outcomes of each clinical study in KT recipients are summarized in Supplementary Table S1.

This lack of paired sequencing data (pre- and post-intervention) represents a critical gap in the literature: it remains unknown whether the administration of prebiotics, probiotics, or synbiotics in the context of KT effectively restores the abundance of butyrate-producing genera or reverses the expansion of Proteobacteria. Existing evidence regarding these alterations comes from observational cohort studies that document post-transplant dysbiosis without evaluating any nutritional intervention [4,5,38]. Bridging this gap—through trials incorporating pre- and post-intervention metagenomic sequencing as a primary outcome across all three categories of biotics—is essential to determine whether clinical benefits are truly explained by direct modulation of the microbiota or result from independent mechanisms.

## 6. Limitations of Current Evidence

The overall interpretation of the presented results must be weighed against significant methodological limitations of the primary studies. A substantial proportion of the available evidence stems from pilot studies conducted at single centers with small patient cohorts. The scarcity of large-scale, randomized, blinded, multicenter clinical trials limits the ability to generalize nutritional recommendations for patients with CKD or KT recipients. Furthermore, there is a lack of uniformity among the therapeutic protocols analyzed; studies differ critically regarding CFU viability and dosage, the taxonomic combinations employed (single-strain vs. multi-species), and—crucially—the timing of therapy initiation (immediate perioperative period vs. late outpatient phase), all of which hinder the establishment of reproducible guidelines.

The study populations are also heterogeneous (non-dialysis CKD, hemodialysis, peritoneal dialysis, and KT recipients at different times after transplantation, with different comorbidities, immunosuppressive regimens, and antibiotic exposure); most interventional studies were conducted in CKD or dialysis patients, and the few trials in KT recipients are small, so extrapolation to the transplant setting is limited.

Most of the few available clinical trials limit follow-up to periods ranging from 4 weeks to 6 months post-intervention. Given that chronic graft rejection, interstitial fibrosis, and tubular atrophy are processes that unfold over years, the absence of long-term studies makes it impossible to definitively confirm whether nutritional modulation of the microbiota translates into improved graft survival at 5 or 10 years. Moreover, studies often analyze the use of biotics as if they were isolated pharmacological interventions, failing to strictly control for patient dietary intake (fiber, protein, dietary patterns, etc.). Since diet is the most potent modulator of the microbiota, the lack of dietary standardization introduces a critical confounding bias when evaluating the efficacy of biotics. Overcoming these limitations will require appropriately powered, multicenter, randomized, placebo-controlled trials with standardized interventions (strain, dose, timing, and duration) and control of dietary intake, incorporating longitudinal shotgun metagenomic sequencing (pre- and post-surgery), microbial metabolomics (e.g., uremic toxins and short-chain fatty acids), intestinal permeability measurements, formal nutritional assessment (including body composition, muscle strength, and dietary intake), and graft-function and clinical outcomes (e.g., rejection, infections, and graft survival). Only then could precision nutrition be considered as a formal adjuvant therapy in international KT guidelines.

This narrative review also has its own limitations. Selection bias cannot be excluded, and relevant studies may have been missed. Publication bias is also likely, since small positive studies are more likely to be published, and some data come from conference abstracts. Finally, the heterogeneity across populations, interventions, and outcomes precluded quantitative synthesis, and findings from CKD or dialysis patients may not apply to KT recipients. Our conclusions should therefore be considered hypothesis-generating.

## 7. Conclusions

The gut microbiota of advanced CKD and KT recipients is profoundly altered: pro-inflammatory and opportunistic taxa (Enterobacteriaceae, *Enterococcus*, Proteobacteria) predominate at the expense of butyrate-producing genera (*Faecalibacterium* and *Roseburia*)—a pattern likely driven by residual solute retention, antibiotic therapy, and chronic immunosuppression. This dysbiosis makes the microbiota a plausible therapeutic target in KT.

Prebiotics appear to be well-tolerated, whereas the safety of probiotics and synbiotics in immunosuppressed patients has not been established. Available evidence, though limited, suggests that these interventions may modify biochemical and digestive parameters associated with dysbiosis. However, the evidence is heterogeneous across populations, comes from small or short studies, and no trial in KT recipients was designed to evaluate nutritional status. Future prospective studies are required to confirm their efficacy and immunological safety, as well as any possible association with infectious adverse events.

## Figures and Tables

**Figure 1:**
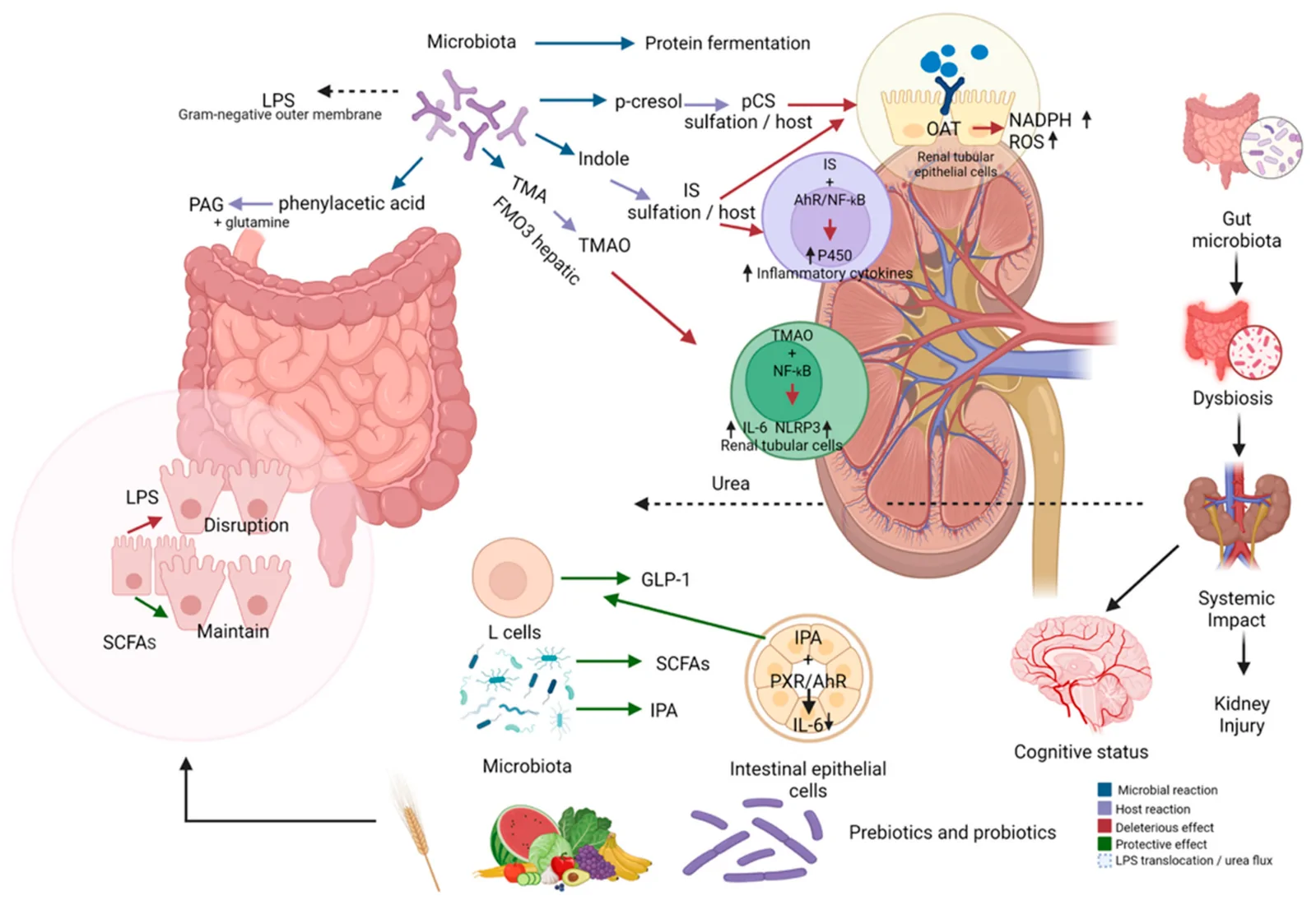
Microbial and host pathways linking gut dysbiosis, uremic toxin generation, intestinal barrier disruption, and renal tubular injury in advanced CKD. Microbial reactions (gut lumen): indole (from tryptophan), p-cresol (from tyrosine and phenylalanine), phenylacetic acid (from phenylalanine), and trimethylamine (TMA, from dietary choline). Host reactions: after absorption, indole and p-cresol are conjugated (sulfation) into indoxyl sulfate (IS) and p-cresyl sulfate (pCS); phenylacetic acid is conjugated with glutamine to form phenylacetylglutamine (PAG); and TMA is oxidized by hepatic flavin-containing monooxygenase 3 (FMO3) into trimethylamine N-oxide (TMAO). Renal tubular cells: IS and pCS enter via the organic anion transporter (OAT), increasing nicotinamide adenine dinucleotide phosphate (NADPH) and reactive oxygen species (ROS); IS activates the aryl hydrocarbon receptor (AhR) and nuclear factor-kappa B (NF-κB), increasing cytochrome P450 (P450) and inflammatory cytokines; TMAO activates NF-κB, increasing interleukin-6 (IL-6) and the nucleotide-binding oligomerization domain, leucine-rich repeat and pyrin domain-containing protein 3 (NLRP3) inflammasome. Intestinal barrier: lipopolysaccharide (LPS), a component of the outer membrane of Gram-negative bacteria (not a fermentation product), disrupts tight junctions and favors endotoxemia. Protective pathways: short-chain fatty acids (SCFAs) help maintain the intestinal barrier; indole-3-propionic acid (IPA) acts through the pregnane X receptor (PXR) and AhR to limit intestinal inflammation and stimulates L cells to release glucagon-like peptide-1 (GLP-1). Prebiotics and probiotics act by modulating the microbiota. Dark-blue arrows: microbial reactions in the gut lumen; light-purple arrows: host reactions (conjugation and oxidation); red arrows: deleterious effects; green arrows: protective effects; dashed arrows: lipopolysaccharide translocation and urea flux.

**Table 1:** Nutritional alterations associated with kidney transplantation, their mechanisms of development, and their clinical relevance.

Alteration	Associated Mechanisms	Clinical Relevance
Persistent PEW	May persist due to residual inflammation, corticosteroid use, and perioperative catabolism	Approximately 20–52% depending on the diagnostic criteria [8].
Post-transplant obesity and weight gain	Corticosteroids and calcineurin inhibitors induce hyperphagia, insulin resistance, and adipokine alterations; this is compounded by improved appetite following the uremia resolution	Obesity (BMI ≥ 30 kg/m^2^): 6.3% before transplantation vs. 18.8% after, in 96 recipients [9]. Weight gain during the first year: 54.6% (body mass index increase ≥2 kg/m^2^) to 72.7% (any weight increase) [10,11].
Post-transplant diabetes mellitus	Corticosteroids and calcineurin inhibitors reduce insulin secretion and sensitivity, exacerbated by post-transplant weight gain	Common metabolic complication that increases cardiovascular risk and can be modified by diet [12]
Hypomagnesemia	Tacrolimus reduces expression of the transient receptor potential cation channel subfamily M Member 6 (TRPM6) in the distal convoluted tubule, increasing magnesium loss	Highly prevalent, it has been linked to increased risk of post-transplant diabetes and alterations in gut microbiota [13]
Gastrointestinal symptoms	Often induced by MMF, which alters gut microbiota and the production of SCFAs, leading to enteropathy and diarrhea	They can compromise intake and adherence to immunosuppressive treatment [14].
Sarcopenia and sarcopenic obesity	Associated to pre-existing kidney disease factors and dialysis-associated chronic inflammation that may persist after transplantation due to immunosuppressives and low physical activity	Pooled prevalence of approximately 26% among recipients (34% when defined by low muscle mass alone and 21% when combined with reduced strength or physical performance) [15,16]; sarcopenic obesity is associated with increased mortality risk, disability, and metabolic disorders [15].

Abbreviations: PEW: protein-energy wasting; MMF: mycophenolate mofetil; SCFAs: short-chain fatty acids.

**Table 3:** Mechanism of action and effect of biotic interventions on gut microbiome composition and on clinical/biochemical outcomes in kidney transplantation.

Intervention	Mechanism of Action	Microbiome Outcomes	Clinical/Biochemical Outcomes
Prebiotics (resistant starch, inulin)	Provision of fermentable substrate favoring indigenous saccharolytic taxa over proteolytic fermentation	Increase in fecal microbial richness (from 96 ± 46 to 142 ± 39; *p* = 0.01) and a trend toward higher Shannon diversity with green banana resistant starch [70]; increase in intestinal Bifidobacterium and SCFA-producing metabolic pathways with inulin [71]	-
Probiotics (*Lactobacillus* spp.)	Competitive exogenous colonization; effect on regulatory T-cell immunomodulation in the mucosa	No RCT of probiotics alone involving microbiota analysis in KT recipients were identified; the only available study is observational and linked probiotic supplementation to a higher incidence of cytomegalovirus viremia, without assessing microbiota [59]	Probiotic supplementation was associated with a higher incidence of cytomegalovirus viremia (observational study) [59]
Synbiotic (*Lactobacillus/Bifidobacterium* + inulin or resistant starch)	Combination of prebiotic substrate and probiotic strain to optimize implantation and persistence	Not assessed in the available trials [78,79]	Significant reduction in plasma pCS [78] and in markers of intestinal permeability and systemic inflammation (LBP, hs-CRP, sICAM-1, IL-6) [79]

SCFAs: short-chain fatty acids; pCS: p-cresyl sulfate; LBP: lipopolysaccharide-binding protein; hs-CRP: high-sensitivity C-reactive protein; IL-6: interleukin-6; sICAM-1: soluble intercellular adhesion molecule-1; KT: kidney transplantation; RCT: randomized controlled trial.

## Data Availability

No new data were created or analyzed in this study. Data sharing is not applicable to this article.

## Supplementary Materials

The following supporting information can be downloaded at: https://www.mdpi.com/article/10.3390/nu18213502/s1. Table S1: Summary of design, sample size, intervention, duration and main outcomes of clinical studies of biotic interventions in kidney transplant recipients.
