# Supplementary material for: Gut Microbiota Dysbiosis in Advanced Chronic Kidney Disease and Kidney Transplantation: A Narrative Review of Pathophysiological Mechanisms, Implications for Nutritional Status, and Biotic Interventions
Source: Nutrients. 2026 Oct 25;18(21):3502. doi: 10.3390/nu18213502 (PMC13764951; doi:10.3390/nu18213502)
Supplement: Supplementary file 1 [file nutrients-18-03502-s001.zip › nutrients-4608600-supplementary.pdf]

Supplementary Table S1. Summary of design, sample size, intervention, duration and main outcomes of clinical studies of biotic interventions in kidney transplant recipients

Study	Design	n	Study Intervention	Duration	Main outcomes
Chan et al. [69]	Randomized, double-blind, placebo-controlled	56 (27 vs. 29)	Green banana resistant starch	7 weeks	Primary: feasibility. Fewer GI symptoms; infections 33% vs. 34%; ↑ fecal richness
Singer et al. [71]	Randomized, double-blind, placebo-controlled	72 (37 vs. 35)	Inulin	4 weeks before and until 4–6 weeks after a third SARS-CoV-2 vaccine dose	Feasible and safe; ↑ Bifidobacterium and SCFA pathways; no improvement in virus neutralization
Guida et al. [78]	Randomized, double-blind, parallel-group (2:1)	34 analyzed (22 vs. 12)	Multi-strain synbiotic	30 days	Plasma p-cresol ↓ 40% (day 15) and 33% (day 30); renal function unchanged; well tolerated
Karimi et al. [79]	Randomized, placebo-controlled	46 analyzed (23 vs. 23)	Synbiotic (12 strains + FOS)	12 weeks	↓ LBP, hs-CRP, sICAM-1, IL-6; weight and intake secondary
Jang et al. [59]	Retrospective, propensity score-matched	354	Probiotics (mean 104.8 days)	Not applicable	CMV viremia 25.8% vs. 14.0% (aOR 2.32)

Abbreviations: pCS, p-cresyl sulfate; LBP, lipopolysaccharide-binding protein; hs-CRP, high-sensitivity C-reactive protein; sICAM-1, soluble intercellular adhesion molecule-1; GI, gastrointestinal; aOR, adjusted odds ratio. Jang et al. is an observational study. No study was designed to evaluate nutritional status as its primary objective.
